# CDX2 and SATB2 loss are associated with myeloid cell infiltration and poor survival in colorectal cancer

**DOI:** 10.1007/s00262-025-03964-x

**Published:** 2025-02-25

**Authors:** Päivi Sirniö, Hanna Elomaa, Anne Tuomisto, Ville K. Äijälä, Henna Karjalainen, Meeri Kastinen, Vilja V. Tapiainen, Onni Sirkiä, Maarit Ahtiainen, Olli Helminen, Erkki-Ville Wirta, Jukka Rintala, Sanna Meriläinen, Juha Saarnio, Tero Rautio, Toni T. Seppälä, Jan Böhm, Jukka-Pekka Mecklin, Markus J. Mäkinen, Juha P. Väyrynen

**Affiliations:** 1 Translational Medicine Research Unit, Medical Research Center Oulu, Oulu University Hospital, and University of Oulu, Aapistie 5A, 90220 Oulu, Finland; 2 Department of Biological and Environmental Science, University of Jyväskylä, Jyväskylä, Finland; 3 Department of Education and Research, Well Being Services County of Central Finland, Jyväskylä, Finland; 4 Department of Pathology, Hospital Nova of Central Finland, Well Being Services County of Central Finland, Jyväskylä, Finland; 5 Department of Environmental and Biological Sciences, University of Eastern Finland, Kuopio, Finland; 6 Department of Gastroenterology and Alimentary Tract Surgery, Tampere University Hospital, Tampere, Finland; 7 Faculty of Medicine and Health Technology, Tampere University and Tays Cancer Centre, Tampere University Hospital, Tampere, Finland; 8 Department of Gastrointestinal Surgery, Helsinki University Central Hospital, University of Helsinki, Helsinki, Finland; 9 Applied Tumor Genomics, Research Program Unit, University of Helsinki, Helsinki, Finland; 10 Faculty of Sport and Health Sciences, University of Jyväskylä, Jyväskylä, Finland

**Keywords:** Colorectal cancer, CDX2, SATB2, Immune cells, Prognosis

## Abstract

**Background:**

Caudal-type homeobox 2 (CDX2) and special AT-rich sequence-binding protein 2 (SATB2) are transcription factors playing important roles in intestinal homeostasis and participating in the regulation of intestinal inflammation. In colorectal cancer (CRC), reduced expression levels of CDX2 and SATB2 have been associated with poor differentiation and worse survival. However, their prognostic significance still needs further clarification, and the associations between CDX2 and SATB2 and immune cell infiltration into the CRC microenvironment are largely unknown.

**Methods:**

We analyzed CDX2 and SATB2 expression in two large cohorts of stages I–IV CRC patients (*N* = 2302) and analyzed their associations with clinicopathologic parameters, the density of local immune cells (determined with three multiplex immunohistochemistry panels and conventional immunohistochemistry), and survival.

**Results:**

In mismatch repair-proficient tumors, reduced CDX2 and SATB2 expression were associated with higher densities of immature monocytic cells, macrophages, and M2-like macrophages. Low expression of CDX2 was associated with shorter cancer-specific survival independent of conventional prognostic parameters in both cohorts. In the larger cohort, adjusted hazard ratio (HR) for negative (vs. high) CDX2 expression was 3.62 (95% CI 2.08–6.31, *p*_trend_ < 0.0001), and adjusted HR for negative (vs. high) SATB2 level was 1.61 (95% CI 0.97–2.67, *p*_trend _= 0.002).

**Conclusion:**

This study indicates that reduced CDX2 and SATB2 expression levels are associated with myeloid cell infiltration in the CRC microenvironment and represent markers for poor outcome. These findings highlight the potential of CDX2 and SATB2 as biomarkers for classifying CRC patients and support their role in regulating the tumor microenvironment.

**Supplementary Information:**

The online version contains supplementary material available at 10.1007/s00262-025-03964-x.

## Introduction

Caudal-type homeobox 2 (CDX2) and special AT-rich sequence-binding protein 2 (SATB2) are transcription factors regulating gene expression in the intestine. CDX2 is normally expressed within the nuclei of intestinal epithelial cells and plays a crucial role in the development and maintenance of the intestinal epithelium [1]. SATB2 is highly expressed in the lower gastrointestinal epithelium and specific neurons in brain and is particularly important in the development of neural, craniofacial, and osteoblastic structures [2]. In the colon, SATB2 is required for maintaining colonic identity of stem cells and differentiated cells [3].

CDX2 and SATB2 have been linked to colorectal cancer (CRC) progression. They both are used as immunohistochemical markers for colorectal origin in tumors and are advantageous in distinguishing metastatic colorectal adenocarcinoma from other adenocarcinomas [4–6]. However, their expression is reduced or lost in a subset of CRCs, most often poorly differentiated CRCs [7, 8]. Reduced expression has been found to be associated with worse survival and several unfavorable prognostic factors, including advanced stage, higher tumor grade, and *BRAF* mutation [8–14]. Decreased CDX2 and SATB2 expression is also associated with mismatch repair (MMR) protein deficiency in CRC [15, 16].

High immune cell infiltration generally predicts better outcome in CRC [17]. The immune cell infiltrate can be quantified using the Immunoscore, a scoring system based on the density of CD3^+^ and CD8^+^ cell populations both at the tumor center and at the invasive margin [18]. A high Immunoscore predicts longer survival, and the prognostic power of the Immunoscore has been validated by a worldwide consortium [19]. In recent years, CDX2 and SATB2 have been increasingly linked to intestinal inflammation. CDX2 has been shown to regulate inflammasome activity through expression of the NLRP3 suppressor TRIM31 [20]. In the mouse colon epithelium, loss of Cdx function leads to elevated infiltration of macrophages and expression of proinflammatory cytokines, including tumor necrosis factor-alpha, interleukin-1 beta, and interleukin-6 [20–22]. Also, the expression of CDX2 and SATB2 has been shown to be decreased in the epithelium of patients with inflammatory bowel disease, and Satb2-deficient mice are more prone to the development of colitis and colitis-associated cancer compared with control mice [23–25]. However, the potential role of CDX2 and SATB2 in the inflammatory response in human CRC is largely unknown.

The primary aim of this study was to evaluate the associations between CDX2 and SATB2 expression and tumor-infiltrating immune cells in CRC. The secondary aim was to confirm the prognostic value of CDX2 and SATB2 in CRC.

## Materials and methods

### Patients

This study was based on two independent cohorts. Cohort 1 was retrospectively collected in Central Finland Central Hospital in Jyväskylä. It included 1343 patients who had undergone tumor resection during 2000–2015 [26]. Cohort 2 was prospectively collected of 1011 newly diagnosed CRC patients operated in the Oulu University Hospital between 2006 and 2020, who had signed an informed consent to participate and were eligible to the study [27]. Patients who had received preoperative radiotherapy or chemoradiotherapy (Cohort 1: *N* = 243; Cohort 2: *N* = 235) were excluded from immune cell analyses, and patients who died within 30 days of initial surgery (Cohort 1: *N* = 40; Cohort 2: *N* = 5) were excluded from survival analysis.

### Immunohistochemistry and image analysis

Immunohistochemistry was conducted on tissue microarrays (TMAs) that were constructed from formalin-fixed paraffin-embedded tissue samples and were designed to contain four cores (diameter 1 mm) per case, two from the center of the tumor (CT) and two from the invasive margin (IM) [26, 27]. MMR enzyme screening status for MLH1, MSH2, MSH6, and PMS2, and *BRAF* V600E mutation status had been previously examined with immunohistochemistry [26, 27]. The immune cell analyses for Cohort 1 were based on multiplex immunohistochemistry and quantitative image analyses. The multiplex immunohistochemistry staining protocol, reagents, and the primary antibodies along with their dilutions and epitope retrieval conditions are demonstrated in Supplementary Fig. S1. Analyses of tumor-infiltrating immune cells were conducted with supervised machine learning approaches built-in QuPath [26, 28, 29]. The cell types investigated in this study included CD3^+^ T cells, CD20^+^CD79A^+^ B cells, CD20^-^CD79A^+^ plasma cells, M1-like and M2-like macrophages, CD14^+^HLA-DR^+^ mature monocytic cells, CD14^+^HLA-DR^-^ immature monocytic cells, CD66B^+^ granulocytes, and tryptase^+^ mast cells. Macrophages were categorized according to their polarization by calculating a polarization index for each macrophage [28]. Immune cell score was calculated according to the principles of Immunoscore® [26].

**Fig. 1:**
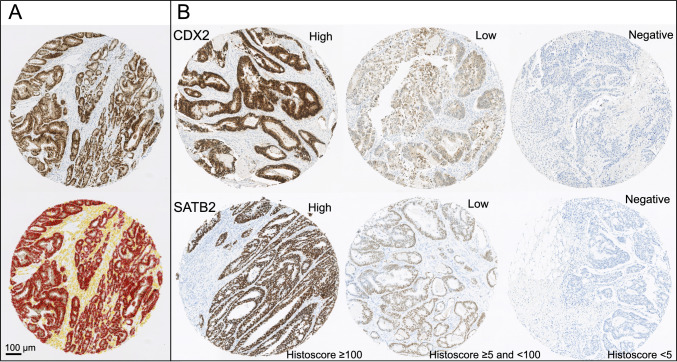
CDX2 and SATB2 immunohistochemistry. **A** Cell detection and classification of one TMA core using QuPath. In the image analysis result image, red color represents cancer cells and yellow other cells. **B** Examples of TMA cores with high, low, and negative CDX2 and SATB2 expression

An automated immunostainer (BOND-III, Leica Biosystems, Germany) and the BOND Refine detection kit (Leica Biosystems) were used for the immunohistochemical staining of CDX2 (Epredia RM-2116, clone EPR2764Y, 1:500) and SATB2 (Cell Marque 384R-16, clone EP281, 1:80). BOND Epitope Retrieval Solution 2 (Leica Biosystems, AR9640) was used in the pretreatment (100 °C, 30 min). The slides were scanned with NanoZoomer XR (Hamamatsu Photonics, Hamamatsu City, Japan) scanner using 20× objective magnification. Scanned images were analyzed with QuPath (version 0.4.4). In QuPath, cell detection function was used to detect cells, add intensity features function was used to calculate Haralick’s texture features, and add smoothed features function was used to calculate smoothed features (within 20 µm). An object classifier was then trained to recognize tumor and stromal cells. The intensity of DAB (3,3’-Diaminobenzidine) chromogen was measured for each tumor cell with QuPath, and the cell data were processed using RStudio and R statistical programming (version 4.3.1, R core Team). For each cell, DAB intensity was classified into four categories. For CDX2, DAB  <  0.25 intensity units indicated negative, DAB 0.25–0.55 weakly positive, DAB 0.55–0.85 moderately positive, and DAB  >  0.85 strongly positive staining. For SATB2, DAB  <  0.2 intensity units indicated negative, DAB 0.2–0.4 weakly positive, DAB 0.4–0.6 moderately positive, and DAB  >  0.6 strongly positive staining. The mean histoscore for each core was calculated using the formula: 1 × percentage of tumor cells showing weak staining + 2 × percentage of tumor cells showing moderate staining + 3 × percentage of tumors cells showing intensive staining. For each tumor, a single histoscore was calculated as the average of histoscores from all individual cores, and tumors were categorized into negative (histoscore < 5), low (histoscore ≥ 5 and < 100), and high expression (histoscore ≥ 100) [30]. Examples of TMA cores with cell classification and different CDX2 and SATB2 expression groups are presented in Fig. 1.

### Statistical analyses

The statistical analyses were conducted using IBM SPSS Statistics for Windows version 29.0.0.0 (IBM Corporation, Armonk, NY, USA). Chi-square test was used to characterize the relationship between categorical variables. Correlations between two continuous variables were determined using the Spearman’s rank correlation test. The 2D visualization was created with Cytoscape software platform [31], utilizing the Prefuse force directed algorithm weighted by the statistical significances of the correlations between individual variables. Survival rates were visualized by the Kaplan–Meier method, and the statistical significance was tested with the log-rank test. Cancer-specific survival (CSS; time from operation to CRC death) was the primary endpoint. Hazard ratios (HRs) with 95% confidence interval (CI) were calculated using Cox proportional hazard models. Multivariable Cox regression models included the following pre-determined covariates: age (< 65, 65–75, and > 75), sex (male and female), stage (I, II, III, and IV), MMR enzyme status (proficient and deficient), *BRAF* V600E mutation status (wild-type and mutant), tumor location (proximal colon, distal colon, and rectum), year of operation (Cohort 1: 2000–2005, 2006–2010, and 2011–2015; Cohort 2: 2006-2010, 2011–2015, and 2016-2020), lymphatic or venous invasion (no and yes), grade (low-grade and high-grade), and preoperative radiotherapy or chemoradiotherapy (no and yes). A two-tailed *p* < 0.01 was considered statistically significant.

## Results

### Distribution of CDX2 and SATB2 staining and association with clinicopathological characteristics

The expression of CDX2 and SATB2 was detected in variable proportions of tumor cell nuclei, while stromal cells were negative for both. The associations of CDX2 and SATB2 histoscore with tumor and patient characteristics are shown in Tables 1 and 2. CDX2 and SATB2 expression could be evaluated from 1329 patients in Cohort 1 and 973 patients in Cohort 2. Of them, 32 (2%) in Cohort 1 and 22 (2%) in Cohort 2 had loss of CDX2 expression, 95 (7%) and 50 (5%) low CDX2 expression and 1202 (90%) and 901 (93%) high CDX2 expression. SATB2 expression was negative in 55 (4%) tumors in Cohort 1 and 120 (12%) tumors in Cohort 2, low in 265 (20%) and 206 (21%) and high in 1009 (76%) and 647 (67%) tumors. In both cohorts, lower CDX2 and SATB2 expression levels were associated with proximal tumor location, high tumor grade, MMR deficiency, and *BRAF* V600E mutation (*p* < 0.001 for all). CDX2 and SATB2 expression groups were associated with one another (*p* < 0.001). In Cohort 1, low expression of CDX2 and SATB2 was also related to advanced stage (*p* = 0.004 and *p* < 0.001, respectively) and lymphovascular invasion (*p* = 0.008 and *p* = 0.002, respectively).

**Table 1:** Associations between CDX2 expression and clinicopathological parameters in colorectal cancers

Characteristic	Cohort 1					Cohort 2				
	Total N	High	Low	Negative	*p*	Total N	High	Low	Negative	*p*
All cases	1329 (100%)	1202 (90%)	95 (7%)	32 (2%)		973 (100%)	901 (93%)	50 (5%)	22 (2%)	
*Sex*										
Female	618 (47%)	548 (46%)	49 (52%)	21 (66%)	0.048	425 (44%)	382 (58%)	29 (58%)	14 (64%)	0.016
Male	711 (54%)	654 (54%)	46 (48%)	11 (34%)		548 (56%)	519 (42%)	21 (42%)	8 (36%)	
*Age (years)*										
<65	395 (30%)	364 (30%)	22 (23%)	9 (28%)	0.243	330 (34%)	303 (34%)	20 (40%)	7 (32%)	0.769
65–75	454 (34%)	415 (35%)	31 (33%)	8 (25%)		356 (37%)	334 (37%)	14 (28%)	8 (36%)	
>75	480 (36%)	423 (35%)	42 (44%)	15 (47%)		287 (29%)	264 (29%)	16 (32%)	7 (32%)	
*Tumor location*										
Proximal colon	532 (40%)	445 (37%)	63 (66%)	24 (75%)	<0.001	321 (33%)	279 (31%)	26 (52%)	16 (73%)	<0.001
Distal colon	399 (30%)	387 (32%)	8 (8%)	4 (13%)		205 (21%)	200 (22%)	3 (6%)	2 (9%)	
Rectum	398 (30%)	370 (31%)	24 (25%)	4 (13%)		447 (46%)	422 (47%)	21 (42%)	4 (18%)	
*Preoperative RT/CRT*										
No Yes	1091 (82%) 239 (18%)	984 (82%) 218 (18%)	77 (81%) 18 (19%)	30 (91%) 3 (9%)	0.397	759 (78%) 214 (22%)	701 (78%) 200 (22%)	39 (78%) 11 (22%)	19 (86%) 3 (14%)	0.632
*AJCC disease stage*										
I	246 (19%)	238 (20%)	7 (7%)	0 (0.0%)	0.004	213 (22%)	202 (22%)	8 (16%)	3 (14%)	0.124
II	485 (37%)	433 (36%)	40 (42%)	13 (41%)		306 (31%)	280 (31%)	18 (36%)	8 (36%)	
III	425 (32%)	382 (32%)	30 (32%)	13 (41%)		346 (36%)	325 (36%)	16 (32%)	5 (23%)	
IV	173 (13%)	149 (12%)	18 (19%)	6 (19%)		108 (11%)	94 (10%)	8 (16%)	6 (27%)	
*Tumor grade*										
Low-grade	1106 (83%)	1051 (87%)	45 (47%)	10 (31%)	<0.001	829 (85%)	793 (88%)	29 (58%)	7 (32%)	<0.001
High-grade	223 (17%)	151 (13%)	50 (53%)	22 (69%)		144 (15%)	108 (12%)	21 (42%)	15 (68%)	
*Lymphovascular invasion*										
No	1043 (78%)	957 (80%)	64 (67%)	22 (69%)	0.008	509 (52%)	478 (53%)	24 (48%)	7 (32%)	0.118
Yes	286 (22%)	245 (20%)	31 (33%)	10 (31%)		464 (48%)	423 (47%)	26 (52%)	15 (68%)	
*MMR status*										
pMMR	1160 (87%)	1083 (90%)	63 (66%)	14 (44%)	<0.001	847 (87%)	809 (90%)	26 (52%)	12 (55%)	<0.001
dMMR	169 (13%)	119 (10%)	32 (34%)	18 (56%)		126 (13%)	92 (10%)	24 (48%)	10 (45%)	
*BRAF status*										
Wild-type	1140 (86%)	1064 (89%)	61 (64%)	15 (47%)	<0.001	862 (89%)	825 (92%)	27 (54%)	10 (45%)	<0.001
Mutant	187 (4%)	136 (11%)	34 (36%)	17 (53%)		111 (11%)	76 (8%)	23 (46%)	12 (55%)	
*SATB2 subgroups*										
High	1009 (76%)	981 (82%)	24 (25%)	4 (13%)	<0.001	647 (67%)	637 (71%)	9 (18%)	1 (5%)	<0.001
Low	265 (20%)	194 (16%)	53 (56%)	18 (56%)		206 (21%)	184 (20%)	20 (40%)	2 (9%)	
Negative	55 (4%)	27 (2%)	18 (19%)	10 (31%)		120 (12%)	80 (9%)	21 (42%)	19 (86%)	

Abbreviations: AJCC, The American Joint Committee on Cancer; RT/CRT, radiotherapy/chemoradiotherapy; MMR, mismatch repair; pMMR, mismatch repair proficient; and dMMR, mismatch repair deficient

**Table 2:** Associations between SATB2 expression and clinicopathological parameters in colorectal cancers

Characteristic	Cohort 1					Cohort 2				
	Total N	High	Low	Negative	*p*	Total N	High	Low	Negative	*p*
All cases	1329 (100%)	1009 (76%)	265 (20%)	55 (4%)		973 (100%)	647 (67%)	206 (21%)	120 (12%)	
*Sex*										
Female	618 (47%)	451 (%)	131 (%)	36 (%)	0.006	425 (44%)	272 (42%)	99 (48%)	54 (45%)	0.302
Male	711 (54%)	558 (%)	134 (%)	19 (%)		548 (56%)	375 (58%)	107 (52%)	66 (55%)	
*Age (years)*										
<65	395 (30%)	299 (30%)	79 (30%)	17 (31%)	0.497	330 (34%)	216 (33%)	73 (35%)	41 (34%)	0.827
65–75	454 (34%)	357 (35%)	81 (31%)	16 (29%)		356 (37%)	240 (37%)	69 (34%)	47 (39%)	
>75	480 (36%)	353 (35%)	105 (40%)	22 (40%)		287 (29%)	191 (30%)	64 (31%)	32 (27%)	
*Tumor location*										
Proximal colon	532 (40%)	352 (35%)	139 (53%)	41 (75%)	<0.001	321 (33%)	179 (28%)	81 (39%)	61 (51%)	<0.001
Distal colon	399 (30%)	338 (34%)	55 (21%)	6 (11%)		205 (21%)	146 (23%)	43 (21%)	16 (13%)	
Rectum	398 (30%)	319 (32%)	71 (27%)	8 (15%)		447 (46%)	322 (50%)	82 (40%)	43 (36%)	
*Preoperative RT/CRT*										
No Yes	1091 (82%) 239 (18%)	822 (82%) 187 (18%)	217 (82%) 49 (18%)	52 (95%) 3 (5%)		759 (78%) 214 (22%)	496 (77%) 151 (23%)	164 (80%) 42 (20%)	99 (83%) 21 (17%)	0.301
*AJCC disease stage*										
I	246 (19%)	212 (21%)	27 (10%)	6 (11%)	<0.001	213 (22%)	155 (24%)	40 (19%)	18 (15%)	0.156
II	485 (37%)	375 (37%)	90 (34%)	21 (38%)		306 (31%)	201 (31%)	64 (31%)	41 (34%)	
III	425 (32%)	306 (30%)	102 (39%)	17 (31%)		346 (36%)	229 (35%)	75 (36%)	42 (35%)	
IV	173 (13%)	116 (12%)	46 (17%)	11 (20%)		108 (11%)	62 (10%)	27 (13%)	19 (16%)	
*Tumor grade*										
Low-grade	1106 (83%)	896 (89%)	181 (68%)	29 (53%)	<0.001	829 (85%)	594 (92%)	153 (74%)	82 (68%)	<0.001
High-grade	223 (17%)	113 (11%)	84 (32%)	26 (47%)		144 (15%)	53 (8%)	53 (26%)	38 (32%)	
*Lymphovascular invasion*										
No	1043 (79%)	813 (81%)	187 (71%)	43 (78%)	0.002	509 (52%)	356 (55%)	102 (50%)	51 (43%)	0.028
Yes	286 (22%)	196 (19%)	78 (29%)	12 (22%)		464 (48%)	291 (45%)	104 (51%)	69 (58%)	
*MMR status*										
pMMR	1160 (87%)	913 (90%)	209 (79%)	38 (69%)	<0.001	847 (87%)	597 (92%)	164 (80%)	86 (72%)	<0.001
dMMR	169 (13%)	96 (10%)	56 (21%)	17 (31%)		126 (13%)	50 (8%)	42 (20%)	34 (28%)	
*BRAF status*										
Wild-type	1140 (86%)	911 (91%)	189 (71%)	40 (73%)	<0.001	862 (89%)	610 (94%)	163 (79%)	89 (74%)	<0.001
Mutant	187 (4%)	96 (10%)	76 (29%)	15 (27%)		111 (11%)	37 (6%)	43 (21%)	31 (26%)	

Abbreviations: AJCC, The American Joint Committee on Cancer; RT/CRT, radiotherapy/chemoradiotherapy; MMR, mismatch repair; pMMR, mismatch repair proficient; and dMMR, mismatch repair deficient

### Association with immune cell densities

**Fig. 2:**
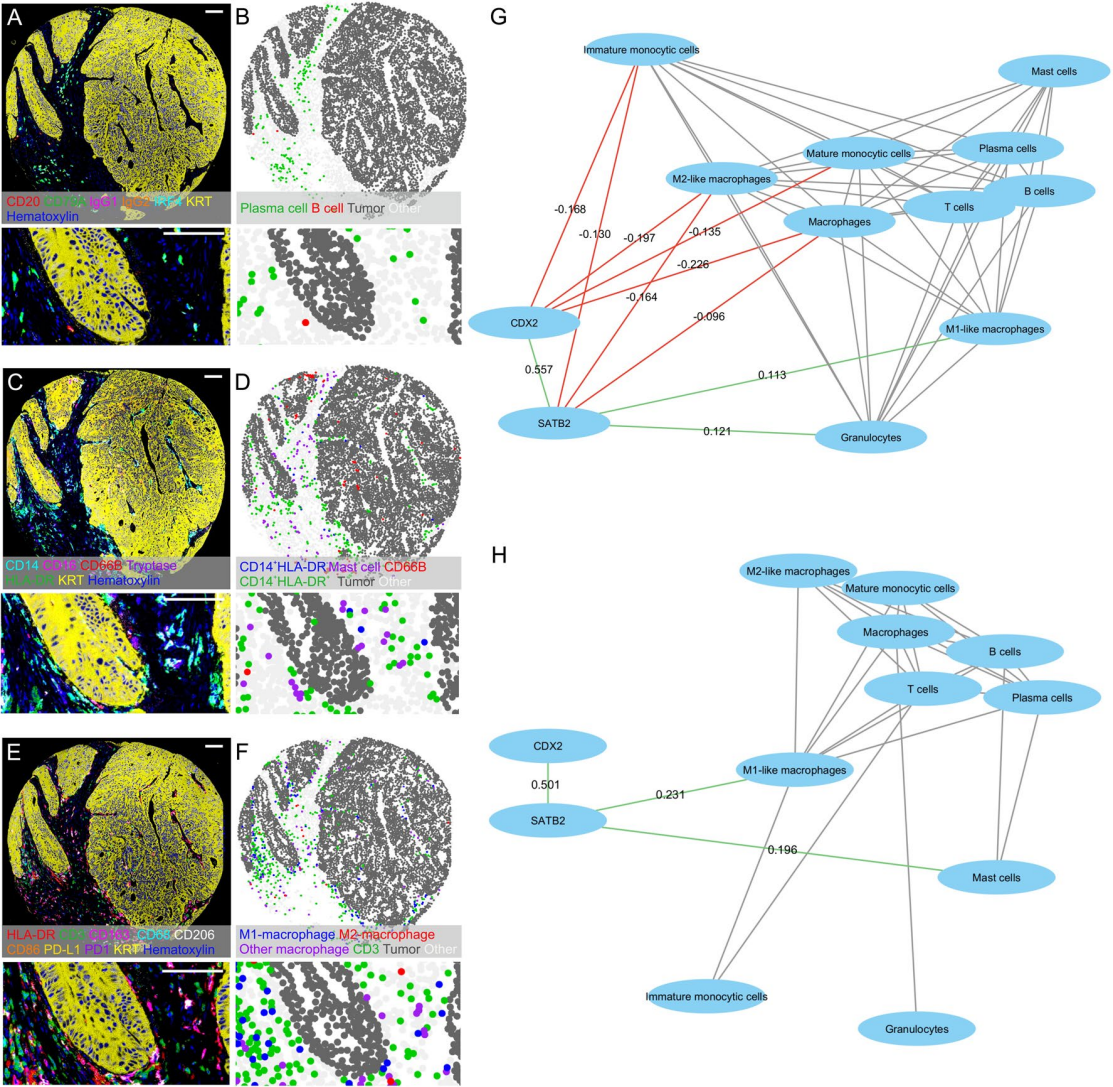
Immune cell analyses. **A**–**F** Multiplex immunohistochemistry images for visualizing three staining panels (**A**, **C**, **E**) and their corresponding cell phenotyping maps (**B**, **D**, **F**) in one tumor core and a close-up view. Scale bars are 100 µm. **G**, **H** Two-dimensional visualization of the relationships between CDX2 and SATB2 expression levels and immune cell densities in mismatch repair-proficient (**G**) and -deficient (**H**) tumors in Cohort 1. The edges (connecting lines) depict the associations between variables (only those with *p* < 0.01 are shown), and the edge length illustrates the significance of the association. The correlations between CDX2 and SATB2 and other variables are represented by green (positive correlation) and red (negative correlation) edges, with the label showing corresponding Spearman correlation coefficient for the correlation. The other associations are represented by gray edges

Next, we investigated whether the expression levels of CDX2 and SATB2 are associated with immune cell infiltration patterns of the tumors. We utilized three multiplex immunohistochemistry panels in Cohort 1 and conducted separate analyses for MMR-proficient (pMMR) and MMR-deficient (dMMR) tumors, considering that both immune cell densities and SATB2 and CDX2 expression are associated with MMR status. Example multiplex immunohistochemistry images of CDX2 high/SATB2 low tumor and their corresponding cell maps are shown in Fig. 2, while images of CDX2 high/SATB2 high tumor and CDX2 negative/SATB2 negative tumor are presented in Supplementary Fig. S2. We found several weak correlations between CDX2 and SATB2 histoscore and the overall densities of different immune cells (Fig. 2, Supplementary Table S2). In pMMR tumors, lower CDX2 histoscore was associated with higher densities of macrophages (*r* = *−*0.226, *p* < 0.001), M2-like macrophages (*r* = *−*0.197, *p* < 0.001), mature monocytic cells (*r* = *−*0.135, *p* < 0.001), and immature monocytic cells (*r* = *−*0.168, *p* < 0.001). Lower SATB2 histoscore was associated with decreased densities of M1-like macrophages (*r* = 0.113, *p* < 0.001) and granulocytes (*r* = 0.121, *p* < 0.001) and increased densities of macrophages (*r* = *−*0.096, *p* = 0.004), M2-like macrophages (*r* = *−*0.164, *p* < 0.001), and immature monocytic cells (*r* = *−*0.130, *p* < 0.001). In dMMR tumors, CDX2 expression showed no significant correlations with immune cells, while lower SATB2 expression was associated with decreased densities of M1-like macrophages (*r* = 0.231, *p* = 0.003) and mast cells (*r* = 0.223, *p* = 0.005). We further analyzed the correlations between CDX2/SATB2 expression and immune cell densities separately at the tumor center and at the invasive margin (Supplementary Table S1), and the findings were largely similar to those for overall immune cell densities. To clarify the effects of the interaction between CDX2 loss and SATB2 loss on immune cell infiltration, we combined CDX2 expression status (high vs. low/negative) with SATB2 expression status (high vs. low/negative). CRCs with both CDX2 and SATB2 low/negative status had significantly lower M1-like macrophage and mast cell densities (Supplementary Fig. S3).

### Survival analyses

**Fig. 3:**
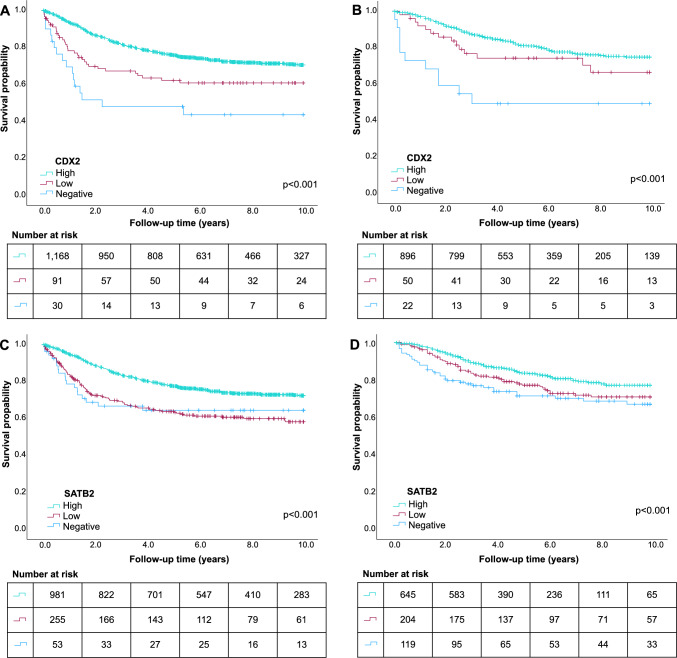
Survival analyses. Kaplan–Meier analysis and log-rank test of colorectal cancer-specific survival according to CDX2 expression in Cohort 1 (**A**) and Cohort 2 (**B**) and SATB2 expression in Cohort 1 (**C**) and Cohort 2 (**D**)

Finally, we analyzed the prognostic value of CDX2 and SATB2 immunohistochemistry. There were 354 cancer deaths in Cohort 1 and 196 in Cohort 2. Kaplan-Meier plots showed that low CDX2 and SATB2 levels predicted shorter CSS compared to high levels in both cohorts (*p* < 0.001 for all, Fig. 3). The prognostic significance of CDX2 remained after adjusting for other confounding factors in multivariable Cox regression models in both cohorts (Table 3, Supplementary Table S2). The multivariable HR for negative (vs. high) CDX2 expression was 3.62 (95% CI 2.08–6.31, *p*_trend_ < 0.0001) in Cohort 1 and 3.55 (95% CI 1.72–7.31, *p*_trend_ < 0.0001) in Cohort 2. For SATB2, a trend of an association between low expression and worse survival was observed in multivariable Cox regression model in Cohort 1 (HR for negative vs. high SATB2 level 1.61, 95% CI 0.97–2.67, *p*_trend_ = 0.002) and a significant association in Cohort 2 (HR for negative vs. high SATB2 level 1.75, 95% CI 1.15–2.67, *p*_trend_ = 0.007) (Table 3, Supplementary Table S2). When we analyzed the survival according to combined CDX2/SATB2 expression category variable, patients with low/negative expression of both CDX2 and SATB2 showed the worst survival outcomes (Supplementary Table S3 and Supplementary Fig. S4).

**Table 3:** Cancer-specific survival according to CDX2 and SATB2 expression in Cohort 1 and 2

	Cohort 1				Cohort 2			
	No. of cases	No. of events	Univariable HR (95% CI)	Multivariable HR (95% CI)	No. of cases	No. of events	Univariable HR (95% CI)	Multivariable HR (95% CI)
*CDX2*								
High	1168	305	1 (referent)	1 (referent)	896	171	1 (referent)	1 (referent)
Low	91	33	1.68 (1.18–2.41)	1.88 (1.23–2.81)	50	14	1.51 (0.87–2.60)	1.25 (0.67–2.32)
Negative	30	16	2.90 (1.75–4.80)	3.62 (2.08–6.31)	22	11	3.86 (2.10–7.10)	3.55 (1.72–7.31)
*p*_trend_			<0.0001	<0.0001			<0.0001	0.003
*SATB2*								
High	981	241	1 (referent)	1 (referent)	645	108	1 (referent)	1 (referent)
Low	255	95	1.82 (1.44–2.31)	1.47 (1.15–1.89)	204	52	1.45 (1.04–2.02)	1.32 (0.93–1.89)
Negative	53	18	1.72 (1.07–2.78)	1.61 (0.97–2.67)	119	36	1.86 (1.28–2.72)	1.75 (1.15–2.67)
*p*_trend_			<0.0001	0.002			0.0005	0.007

Multivariable Cox proportional hazards regression models were adjusted for sex, age (< 65, 65–75, and > 75), year of operation (Cohort 1: 2000–2005, 2006–2010, and 2011–2015, Cohort 2: 2006–2010, 2011–2015, and 2016–2020), tumor location (proximal colon, distal colon, and rectum), disease stage (I, II, III, and IV), tumor grade (well/moderately differentiated and poorly differentiated), lymphovascular invasion (negative and positive), MMR status (proficient and deficient), *BRAF* status (wild-type and mutant), and preoperative radiotherapy or chemoradiotherapy (no and yes)

*p*_trend_ values were calculated by using the three ordinal categories of CDX2 histoscore and SATB2 histoscore as continuous variables in univariable and multivariable Cox proportional hazard regression models

CI confidence interval and HR hazard ratio

To compare the prognostic value of CDX2 and SATB2 histoscore and immune cell score, we included these variables in the same multivariable Cox regression models (Supplementary Table S4). In these models, CDX2 histoscore had prognostic value in both cohorts (*p*_trend_ < 0.0001 in Cohort 1 and *p*_trend_ = 0.0005 in Cohort 2) independently of immune cell score (*p*_trend_ = 0.001 in Cohort 1 and *p*_trend_ = 0.002 in Cohort 2). Additionally, CDX2/SATB2 combination variable had prognostic value independent of immune cell score. The prognostic value of SATB2 histoscore remained in the multivariable model including immune cell score in Cohort 1 (*p*_trend_ = 0.0004), but not in Cohort 2 (*p*_trend_ = 0.019). Subgroup analyses were performed to further assess the prognostic significance of CDX2 and SATB2 expression status according to various clinicopathologic features (Supplementary Fig. S6), but no consistent findings of a significant difference according to any clinicopathologic characteristic were discovered across both cohorts.

## Discussion

We evaluated the associations of CDX2 and SATB2 expression with immune cell infiltration and prognosis in two large CRC cohorts. We found that reduced CDX2 and SATB2 expression were associated with increased infiltration of myeloid cells, most prominently immature monocytic cells and M2-like macrophages, in pMMR tumors, while reduced SATB2 expression in dMMR tumors was associated with lower infiltration of M1-like macrophages and mast cells. Lower CDX2 expression predicted worse CSS in both cohorts independent of conventional prognostic parameters and immune cell infiltrates. These findings underscore the potential of CDX2 and SATB2 as biomarkers for stratifying CRC patients and highlight their significance in regulating the tumor microenvironment, which could inform the development of targeted therapies.

As CDX2 and SATB2 have been linked to intestinal inflammation, we hypothesized that loss of CDX2 and SATB2 expression might be associated with alterations in immune cell infiltration patterns in CRC. We found that both lower CDX2 and SATB2 expression were associated with increased macrophage, M2-like macrophage, and immature monocytic cell infiltrates in pMMR tumors. In addition, lower CDX2 expression was associated with increased mature monocytic cell densities and lower SATB expression with decreased M1-like macrophage and granulocyte densities. In dMMR tumors, lower SATB2 expression was associated with decreased densities of M1-like macrophages and mast cells. In combined analyses that included both CDX2 and SATB2 status, CRCs with low/negative expression of both CDX2 and SATB2 had significantly lower densities of M1-like macrophages and mast cells. Although the correlations were weak, these findings provide new insights into the potential role of CDX2 and SATB2 in CRC-associated inflammatory reactions. Previously, only a few studies have examined the associations between tumor-infiltrating immune cells and the expression of CDX2 or SATB2 in CRC. Chewchuk et al. [32] demonstrated that loss of Cdx function leads to attenuation of histocompatibility complex protein H2-T3, followed by a decrease in iCD8α cell number and degranulation, and an increase in macrophage infiltration in the murine intestinal epithelium. Our results align with this finding, as CDX2 expression showed a negative correlation with macrophage densities. Wang et al. have also reported a negative association between the expression of CDX2 and tumor-infiltrating macrophages [22]. However, they also showed a negative association between CDX2 expression and T-cell infiltrate, whereas we found no correlation between T-cell density and CDX2 or SATB2 expression status. Ni et al. [23] studied colitis model in intestinal epithelial-specific Satb2 knockout mice and control mice, and observed higher numbers of M1 macrophages in Satb2 knockout mice. In contrast with this, we found that lower SATB2 expression was associated with decreased M1-like macrophage infiltrate. Our results suggest that CDX2 and SATB2 expression could affect myeloid cell recruitment and macrophage balance (M1 vs. M2). While the underlying mechanisms remain hypothetical, it is possible that loss of CDX2/SATB2 expression leads to increased expression of cytokines that drive macrophage polarization toward M2 phenotype [21]. Additionally, the associations with altered immune cell densities might be related to disruption of the intestinal epithelial barrier, as CDX2 is essential for maintaining its integrity and function [1]. Compared to the previous studies, our analyses benefited from the use of multiplex immunohistochemistry that enabled more precise detection of different immune cell types. For example, there are no single specific markers for macrophage polarization states [33], and our method was based on calculating a polarization index based on the expression of four polarization markers.

Reduced CDX2 expression was an independent factor for worse CSS in multivariable analysis in both cohorts, whereas reduced SATB2 expression was independent prognostic factor in one cohort and showed a trend of an association in the other cohort. Previously, CDX2 loss has been identified as an independent poor prognostic factor in both non-metastatic and metastatic CRC [9, 11, 13, 22, 34, 35] although not all studies have confirmed its prognostic value [36, 37]. Independent prognostic significance of SATB2 expression has also been reported [8, 10, 35]. Schmitt et al. [8] previously reported a higher prognostic value for SATB2 loss compared to CDX2 loss, whereas we found that the prognostic impact of CDX2 loss was higher compared to SATB2 loss. Lee et al. [38] analyzed concomitant expression patterns of CDX2 and SATB2 as prognostic factors and found that concomitant loss of CDX2 and SATB2, but not isolated loss of CDX2 or SATB2, is a prognostic marker for stage III CRCs. The prognostic significance of CDX2 and SATB2 may be different in specific subgroups such as pMMR and dMMR tumors. Most studies have reported a prognostic value for CDX2 in pMMR tumors [12, 14, 34, 39], while Ma et al. [35] reported an impact only in patients with dMMR but not pMMR tumors. In our subgroup analyses, we found no statistically significant difference in the prognostic significance of CDX2 or SATB2 according to MMR status. Considering that CDX2 and SATB2 expression were associated with immune cell infiltration patterns, we also compared their prognostic value with the immune cell score. In this analysis, the prognostic significance of CDX2 and CDX2/SATB2 combination variable remained independent of immune cell score in both cohorts.

The frequency of CDX2 loss was 2% and SATB2 loss 4–12% in the two cohorts. These findings are in line with the previous studies reporting CDX2 loss in 1.3–5.9% and SATB2 loss in 6.6–28.8% of CRCs [8–10, 40, 41]. These results support the applicability of both transcription factors as markers of tumors with colorectal origin. When applying these markers in the diagnostics of tumors of uncertain origin, it needs to be noted that loss of CDX2 and SATB2 expression was associated with higher tumor grade, proximal tumor location, *BRAF* mutation, and dMMR, which also corresponds with the findings of the previous studies [7, 15, 36, 41, 42]. While CDX2 appears to have somewhat higher sensitivity for CRC than SATB2, the previous studies have indicated that SATB2 has higher specificity for CRC, as CDX2 is frequently also expressed by adenocarcinomas of the pancreas or the upper gastrointestinal tract among others [5, 43].

This study has some limitations. First, protein expression is heterogeneous in tumor tissue, and we used TMAs, which only represent small areas of the tumor. However, the previous studies have shown the applicability of TMAs in the analysis of immune cell infiltrates in CRC [44, 45]. Moreover, CDX2 and SATB2 expression generally showed little variation between cores sampled from different parts of the tumors. Second, data on cancer adjuvant treatments were not available, and we could not assess whether CDX2 or SATB2 expression would predict response to specific treatments. The strengths of this study include the utilization of two large CRC cohorts that contained extensive clinicopathologic data. The immune cell analyses were stratified by MMR status which is an important confounding factor that is associated with both immune cell densities and CDX2/SATB2 expression. Supervised machine learning technique facilitated quantitative image analysis and provided more accurate estimates of protein expression levels in comparison with visual evaluation.

In conclusion, reduced CDX2 and SATB2 expression are associated with increased infiltration of myeloid cells in pMMR tumors and exhibit unfavorable prognostic effect in CRC. These findings support the role of CDX2 and SATB2 in regulating the tumor microenvironment, as well as their potential as prognostic biomarkers in CRC.

## Supplementary Information

Below is the link to the electronic supplementary material.

Supplementary file1 (PDF 1436 KB)

## Data Availability

The data generated and/or analyzed during this study are not publicly available. The sharing of data will require approval from relevant ethics committees and/or biobanks. Further information including the procedures to obtain and access data of Finnish Biobanks are described at https://finbb.fi/en/fingenious-service.

## Abbreviations

AJCC: The American Joint Committee on Cancer
CDX2: Caudal-type homeobox 2
CI: Confidence interval
CRC: Colorectal cancer
CSS: Cancer-specific survival
CT: Center of the tumor
dMMR: Mismatch repair deficient
HR: Hazard ratio
IM: Invasive margin
MMR: Mismatch repair
pMMR: Mismatch repair proficient
RT/CRT: Radiotherapy/chemoradiotherapy
SATB2: Special AT-rich sequence-binding protein 2
TMA: Tissue microarray
