# Supplementary material for: CDX2 and SATB2 loss are associated with myeloid cell infiltration and poor survival in colorectal cancer
Source: Cancer Immunol Immunother. 2025 Feb 25;74(4):111. doi: 10.1007/s00262-025-03964-x (PMC11861821; doi:10.1007/s00262-025-03964-x)
Supplement: Supplementary file 1 — Supplementary file1 (PDF 1436 KB) [file 262_2025_3964_MOESM1_ESM.pdf]

CDX2 and SATB2 loss are associated with myeloid cell infiltration and poor survival in colorectal cancer

Sirniö et al.

Supplementary material

Table S1. Associations of CDX2 and SATB2 histoscore with tumor infiltrating immune cells in Cohort 1.

Variable	CDX2						SATB2					
	dMMR			pMMR			dMMR			pMMR		
	N	r	p	N	r	p	N	r	p	N	r	p
T cells overall	161	-0.053	0.506	899	-0.067	0.045	161	0.079	0.320	899	0.078	0.020
T cells CT	157	-0.100	0.213	881	-0.016	0.627	157	0.027	0.734	881	0.109	0.001
T cells IM	147	0.044	0.594	810	-0.096	0.006	147	0.076	0.357	810	0.044	0.213
B cells overall	165	0.012	0.879	902	-0.062	0.061	165	0.100	0.200	902	0.022	0.513
B cells CT	161	-0.041	0.607	888	0.027	0.427	161	0.040	0.612	888	0.095	0.005
B cells IM	151	0.029	0.723	806	-0.131	<0.001	151	0.079	0.338	806	-0.014	0.694
Plasma cells overall	165	-0.031	0.690	902	-0.031	0.350	165	0.003	0.972	902	0.038	0.248
Plasma cells CT	161	0.011	0.894	888	-0.010	0.766	161	-0.023	0.070	888	0.068	0.044
Plasma cells IM	151	-0.036	0.657	806	-0.100	0.005	151	-0.001	0.993	806	-0.004	0.908
Macrophages overall	161	-0.076	0.337	899	-0.226	<0.001	161	0.033	0.675	899	-0.096	0.004
Macrophages CT	157	-0.109	0.172	881	-0.179	<0.001	157	0.011	0.890	881	-0.083	0.014
Macrophages IM	147	0.017	0.836	810	-0.212	<0.001	147	-0.017	0.838	810	-0.075	0.032
M1-like macrophages overall	161	0.168	0.033	899	-0.007	0.834	161	0.231	0.003	899	0.113	<0.001
M1-like macrophages CT	157	0.197	0.013	881	-0.010	0.770	157	0.213	0.007	881	0.099	0.003
M1-like macrophages IM	147	0.141	0.089	810	0.004	0.899	147	0.118	0.154	810	0.111	0.001
M2-like macrophages overall	161	-0.130	0.101	899	-0.197	<0.001	161	-0.065	0.415	899	-0.164	<0.001
M2-like macrophages CT	157	-0.213	0.007	881	-0.163	<0.001	157	-0.121	0.131	881	-0.142	<0.001
M2-like macrophages IM	147	-0.028	0.732	810	-0.197	<0.001	147	-0.042	0.611	810	-0.139	<0.001
Mature monocytic cells overall	158	-0.153	0.056	883	-0.135	<0.001	158	-0.073	0.359	883	-0.039	0.248
Mature monocytic cells CT	151	-0.106	0.195	846	-0.116	<0.001	151	-0.057	0.489	846	-0.048	0.166
Mature monocytic cells IM	140	-0.134	0.113	762	-0.153	<0.001	140	-0.142	0.095	762	-0.031	0.399
Immature monocytic cells overall	158	-0.048	0.549	883	-0.168	<0.001	158	-0.107	0.182	883	-0.130	<0.001
Immature monocytic cells CT	151	0.026	0.752	846	-0.173	<0.001	151	-0.011	0.896	846	-0.171	<0.001
Immature monocytic cells IM	140	-0.075	0.378	762	-0.152	<0.001	140	-0.124	0.144	762	-0.097	0.007
Granulocytes overall	158	-0.170	0.033	883	-0.042	0.214	158	0.049	0.544	883	0.121	<0.001
Granulocytes CT	151	-0.142	0.081	846	-0.046	0.186	151	0.021	0.801	846	0.080	0.020
Granulocytes IM	140	-0.131	0.124	762	-0.042	0.249	140	0.062	0.464	762	0.142	<0.001
Mast cells overall	158	0.196	0.014	883	-0.010	0.763	158	0.223	0.005	883	0.069	0.039
Mast cells CT	151	0.110	0.180	846	-0.014	0.691	151	0.116	0.156	846	0.047	0.175
Mast cells IM	140	0.230	0.006	762	-0.037	0.311	140	0.205	0.015	762	0.072	0.048

Abbreviations: dMMR, mismatch repair deficient; CT, center of the tumor; IM, invasive margin; pMMR, mismatch repair proficient.

Table S2. Multivariable Cox regression models for cancer-specific survival in Cohort 1 and Cohort 2.

	CDX2 models		SATB2 models	
	**Cohort 1**	**Cohort 2**	**Cohort 1**	**Cohort 2**
	**Multivariable HR (95% CI)**	**Multivariable HR (95% CI)**	**Multivariable HR (95% CI)**	**Multivariable HR (95% CI)**
CDX2			–	–
High	1 (referent)	1 (referent)		
Low	1.88 (1.23–2.81)	1.51 (0.87–2.60)		
Negative	3.62 (2.08–6.31)	3.86 (2.10–7.10)		
SATB2	–	–		
High			1 (referent)	1 (referent)
Low			1.47 (1.15–1.89)	1.45 (1.04–2.02)
Negative			1.61 (0.97–2.67)	1.86 (1.28–2.72)
Year of operation				
2000–2005	1 (referent)		1 (referent)	
2006–2010	0.64 (0.49–0.83)	1 (referent)	0.60 (0.46–0.77)	1 (referent)
2011–2015	0.52 (0.40–0.67)	0.83 (0.58–1.20)	0.52 (0.40–0.67)	0.81 (0.57–1.17)
2016–2020		0.60 (0.41–0.87)		0.62 (0.43–0.90)
Age				
<65	1 (referent)	1 (referent)	1 (referent)	1 (referent)
65–75	1.15 (0.88–1.51)	1.67 (1.17–2.38)	1.22 (0.93–1.59)	1.68 (1.17–2.39)
>75	1.96 (1.50–2.56)	2.62 (1.80–3.80)	2.06 (1.58–2.70)	2.81 (1.92–4.13)
Sex				
Male	1 (referent)	1 (referent)	1 (referent)	1 (referent)
Female	0.87 (0.70–1.08)	0.99 (0.74–1.33)	0.88 (0.70–1.09)	0.98 (0.73–1.31)
Tumor location				
Proximal colon	1 (referent)	1 (referent)	1 (referent)	1 (referent)
Distal colon	0.97 (0.74–1.27)	1.15 (0.75–1.74)	0.93 (0.71–1.22)	1.11 (0.73–1.69)
Rectum	0.87 (0.60–1.26)	0.96 (0.63–1.44)	0.81 (0.56–1.17)	0.91 (0.60–1.37)
Preoperative radiotherapy or chemoradiotherapy				
No	1 (referent)	1 (referent)	1 (referent)	1 (referent)
Yes	1.11 (0.74–1.67)	1.16 (0.78–1.72)	1.18 (0.78–1.77)	1.26 (0.85–1.90)
Disease stage				
I	1 (referent)	1 (referent)	1 (referent)	1 (referent)
II	1.67 (0.99–2.82)	1.54 (0.71–3.33)	1.75 (1.03–2.95)	1.53 (0.70–3.32)
III	4.37 (2.64–7.22)	3.92 (1.90–8.11)	4.41 (2.67–7.29)	3.85 (1.86–7.97)
IV	29.56 (17.67–49.45)	21.65 (10.25–45.75)	29.51 (17.63–49.39)	22.43 (10.60–47.47)
Tumor grade				
Low-grade	1 (referent)	1 (referent)	1 (referent)	1 (referent)
High-grade	1.52 (1.14–2.03)	1.45 (1.00–2.09)	1.65 (1.25–2.18)	1.33 (0.92–1.93)
Lymphovascular invasion				
No	1 (referent)	1 (referent)	1 (referent)	1 (referent)
Yes	1.87 (1.49–2.35)	2.11 (1.44–3.09)	1.88 (1.50–2.36)	2.11 (1.44–3.09)
MMR status				
MMR proficient	1 (referent)	1 (referent)	1 (referent)	1 (referent)
MMR deficient	0.39 (0.24–0.64)	0.47 (0.23–0.97)	0.47 (0.30–0.77)	0.44 (0.21–0.90)
BRAF status				
Wild-type	1 (referent)	1 (referent)	1 (referent)	1 (referent)
Mutant	1.45 (0.98–2.14)	1.10 (0.60–2.00)	1.31 (0.88–1.95)	1.35 (0.79–2.31)

Abbreviations: CI, confidence interval; HR, hazard ratio; MMR, mismatch repair.

Table S3. Cancer-specific survival according to combinatory status of CDX2 and SATB2 expression in Cohort 1 and 2.

	Cohort 1				Cohort 2			
	No. of cases	No. of events	Univariable HR (95% CI)	Multivariable HR (95% CI)	No. of cases	No. of events	Univariable HR (95% CI)	Multivariable HR (95% CI)
**CDX2/SATB2**								
Both H	953	232	1 (referent)	1 (referent)	635	106	1 (referent)	1 (referent)
CDX2 H, SATB2 L/N	215	73	1.63 (1.25−2.12)	1.23 (0.94−1.62)	261	65	1.42 (1.04−1.94)	1.27 (0.90−1.78)
CDX2 L/N, SATB2 H	28	9	1.55 (0.80−3.02)	1.26 (0.63−2.55)	10	2	1.33 (0.33−5.37)	0.35 (0.74−1.69)
Both L/N	93	40	2.35 (1.68−3.29)	2.94 (1.99−4.33)	62	23	2.49 (1.58−3.90)	2.34 (1.37−4.00)
p			<0.001	<0.001			<0.001	0.004

Multivariable Cox proportional hazards regression models were adjusted for sex, age (<65, 65–75, >75), year of operation (cohort 1: 2000–2005, 2006–2010, 2011–2015, cohort 2: 2006-2010, 2011-2015, 2016-2020), tumor location (proximal colon, distal colon, rectum), disease stage (I, II, III, IV), tumor grade (well/moderately differentiated, poorly differentiated), lymphovascular invasion (negative, positive), MMR status (proficient, deficient), *BRAF* status (wild-type, mutant) and preoperative radiotherapy or chemoradiotherapy (no, yes).
CI confidence interval, H High, HR hazard ratio, L/N Low/Negative.

Table S4. Comparison of prognostic power of CDX2 and SATB2 expression and immune cell score using Cox regression models for cancer-specific survival.

	No. of cases	No. of events	Model 1 (univariable) HR (95% CI)	Model 2 (multivariable) HR (95% CI)	Model 3 (multivariable) HR (95% CI)
**Cohort 1**					
CDX2					
High	1,068	285	1 (referent)	1 (referent)	1 (referent)
Low	76	30	1.86 (1.27–2.72)	1.92 (1.31–2.82)	2.35 (1.56–3.55)
Negative	29	15	2.91 (1.73–4.89)	3.55 (2.10–6.00)	4.61 (2.60–8.18)
p_{trend}			<0.0001	<0.0001	<0.0001
Immune cell score					
0	232	86	1 (referent)	1 (referent)	1 (referent)
1	692	203	0.75 (0.58–0.97)	0.75 (0.58–0.97)	0.77 (0.59–1.00)
2	249	41	0.41 (0.28–0.60)	0.38 (0.26–0.55)	0.52 (0.35–0.77)
p_{trend}			<0.0001	<0.0001	0.001
SATB2					
High	900	224	1 (referent)	1 (referent)	1 (referent)
Low	225	88	1.85 (1.44–2.38)	1.77 (1.38–2.28)	1.59 (1.22–2.06)
Negative	48	18	1.99 (1.23–3.21)	2.24 (1.38–3.62)	2.08 (1.25–3.44)
p_{trend}			<0.0001	<0.0001	0.0004
Immune cell score					
0	232	86	1 (referent)	1 (referent)	1 (referent)
1	692	203	0.75 (0.58–0.97)	0.78 (0.60–1.01)	0.78 (0.60–1.01)
2	249	41	0.41 (0.28–0.60)	0.42 (0.29–0.61)	0.56 (0.38–0.83)
p_{trend}			<0.0001	<0.0001	0.003
CDX2/SATB2					
Both H	878	214	1 (referent)	1 (referent)	1 (referent)
CDX2 H, SATB2 L/N	190	63	1.57 (1.19–2.08)	1.50 (1.13–1.99)	1.20 (0.90–1.61)
SATB2 H, CDX2 L/N	22	5	1.06 (0.44–2.57)	1.10 (0.45–2.66)	0.86 (0.34–2.17)
Both L/N	83	39	2.72 (1.93–3.83)	2.94 (2.08–4.14)	3.48 (2.32–5.20)
p			<0.0001	<0.0001	<0.0001
Immune cell score					
0	232	86	1 (referent)	1 (referent)	1 (referent)
1	692	203	0.75 (0.58–0.97)	0.79 (0.61–1.03)	0.80 (0.61–1.04)
2	249	41	0.41 (0.28–0.60)	0.40 (0.28–0.59)	0.53 (0.36–0.79)
p_{trend}			<0.0001	<0.0001	0.002
**Cohort 2**					
CDX2					
High	878	167	1 (referent)	1 (referent)	1 (referent)
Low	50	14	1.51 (0.88–2.61)	1.56 (0.90–2.69)	1.46 (0.78–2.71)
Negative	21	11	4.14 (2.25–7.63)	5.58 (2.98–10.45)	4.05 (1.95–8.42)
p_{trend}			<0.0001	<0.0001	0.0005
Immune cell score					
0	187	74	1 (referent)	1 (referent)	1 (referent)
1	534	96	0.41 (0.30–0.55)	0.43 (0.32–0.59)	0.67 (0.49–0.93)
2	228	22	0.22 (0.14–0.35)	0.19 (0.12–0.31)	0.45 (0.26–0.78)
p_{trend}			<0.0001	<0.0001	0.002
SATB2					
High	631	105	1 (referent)	1 (referent)	1 (referent)
Low	200	51	1.46 (1.04–2.04)	1.32 (0.94–1.85)	1.28 (0.88–1.86)
Negative	118	36	1.91 (1.31–2.79)	1.75 (1.20–2.56)	1.66 (1.08–2.54)
p_{trend}			0.0003	0.003	0.019
Immune cell score					
0	187	74	1 (referent)	1 (referent)	1 (referent)
1	534	96	0.41 (0.30–0.55)	0.42 (0.31–0.58)	0.74 (0.53–1.03)
2	228	22	0.22 (0.14–0.35)	0.23 (0.14–0.37)	0.55 (0.32–0.94)
p_{trend}			<0.0001	<0.0001	0.017
CDX2/SATB2					
Both H	621	103	1 (referent)	1 (referent)	1 (referent)
CDX2 H, SATB2 L/N	257	64	1.44 (1.05–1.97)	1.29 (0.94–1.77)	1.21 (0.85–1.72)
SATB2 H, CDX2 L/N	10	2	1.34 (0.33–5.44)	1.82 (0.45–7.40)	0.47 (0.10–2.72)
Both L/N	61	23	2.55 (1.62–4.01)	2.55 (1.61–4.03)	2.47 (1.45–4.22)
p			<0.0001	<0.0001	0.004
Immune cell score					
0	187	74	1 (referent)	1 (referent)	1 (referent)

1	534	96	0.41 (0.30–0.55)	0.43 (0.32–0.59)	0.71 (0.51–1.00)
2	228	22	0.22 (0.14–0.35)	0.22 (0.13–0.35)	0.54 (0.31–0.94)
p_{trend}			<0.0001	<0.0001	0.013

Model 2: Cox proportional hazards regression model including CDX2 or SATB2 expression and immune cell score.
Model 3: Cox proportional hazards regression model based on Model 2 that was additionally adjusted for sex, age (<65, 65–75, >75), year of operation (cohort 1: 2000–2005, 2006–2010, 2011–2015, cohort 2: 2006-2010, 2011-2015, 2016-2020), tumor location (proximal colon, distal colon, rectum), disease stage (I, II, III, IV), tumor grade (well/moderately differentiated, poorly differentiated), lymphovascular invasion (negative, positive), MMR status (proficient, deficient), *BRAF* status (wild-type, mutant) and preoperative radiotherapy or chemoradiotherapy (no, yes).
P_{trend} values were calculated by using the three ordinal categories of CDX2 histoscore, SATB2 histoscore and immune cell score as continuous variables in univariable and multivariable Cox proportional hazard regression models.
CI confidence interval, HR hazard ratio, H High, L/N Low/Negative.

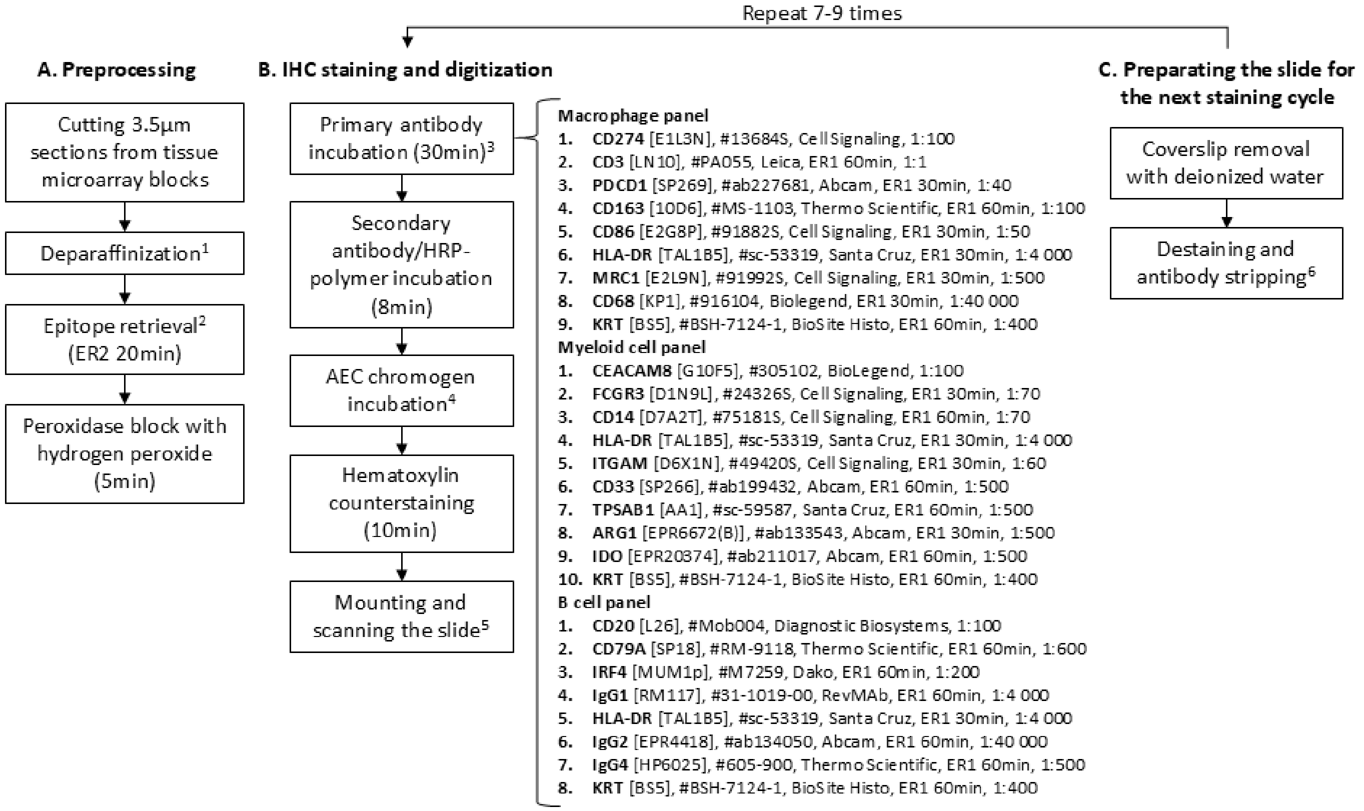

Figure S1. Multiplex immunohistochemistry protocol. Tissue microarray slides were stained 8 (B cell panel), 9 (Macrophage panel), or 10 (myeloid cell panel) times sequentially using an automated immunohistochemistry stainer. The incubations were performed in room temperature if not otherwise specified. Abbreviations: IHC, immunohistochemistry; AEC, 3-Amino-9-ethylcarbazole

1 Dewax solution (AR9222, Leica Biosystems), 30min, 60–72 ˚C.

2 Epitope retrieval done with BOND epitope retrieval solution 2 (EDTA based, pH 9, AR9640, Leica Biosystems) with 20min heating time.

3 Primary antibodies (name, clone, catalogue number and manufacturer) together with the selected antigen retrieval conditions and used dilutions are listed in the staining order.

4 AEC + high sensitivity substrate (K3469, Dako).

5 VectaMount AQ Aqueous Mounting Medium (H-5501, Vector Laboratories), digitized with a 20x objective using NanoZoomer XR (Hamamatsu) slide scanner.

6 The sections were destained with ethanol and antibody stripping was done with heat-induced epitope retrieval using BOND epitope retrieval solution 1 (citrate based, pH 6, AR9961, Leica Biosystems) or 2. Suitable epitope retrieval conditions were optimized for each antibody.

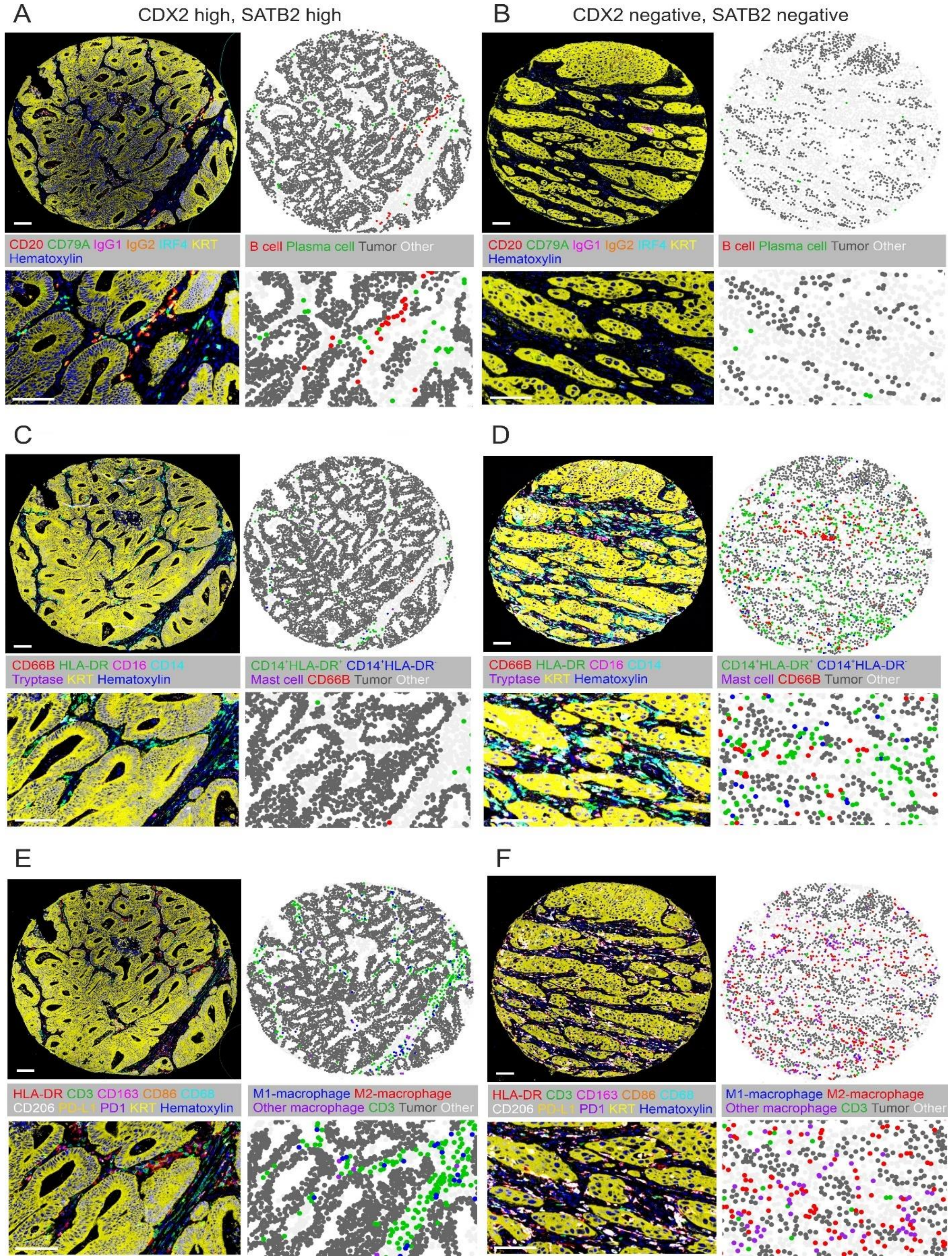

Figure S2. Example multiplex immunohistochemistry images of CDX2 high/SATB2 high (A,C,E) and CDX2 negative/SATB2 negative (B,D,F) tumors, their corresponding cell maps, and close-up views.

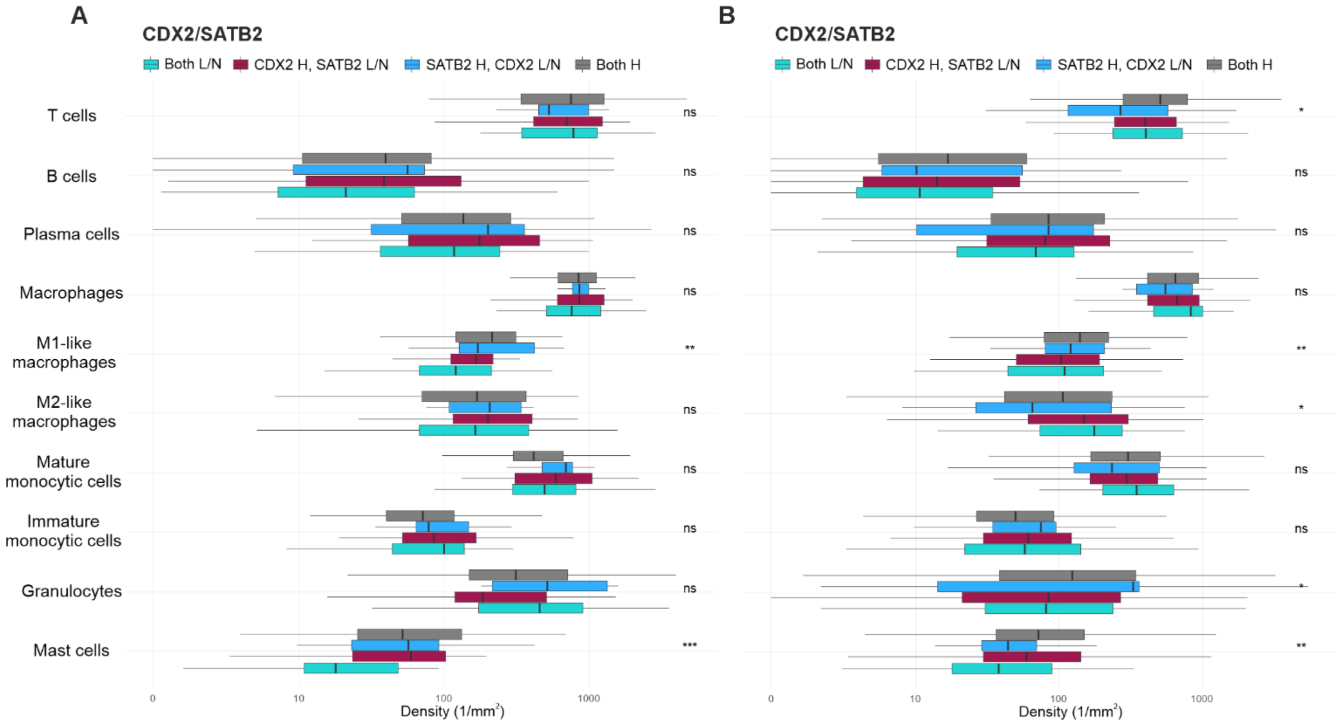

Figure S3. Boxplots of distributions of immune cell densities in the mismatch repair deficient tumors (A) and mismatch repair proficient tumors (B) according to combinatory status of CDX2 and SATB2 expression in Cohort 1. *p value<0.05, **p value<0.01, ***p value<0.001. H High, L/N Low/Negative.

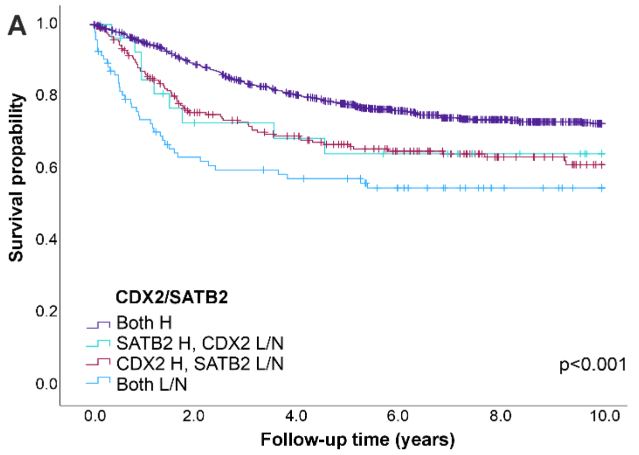

Number at risk

	953	804	685	533	399	276
	28	18	16	14	11	7
	215	146	123	98	67	51
	93	53	47	39	27	23

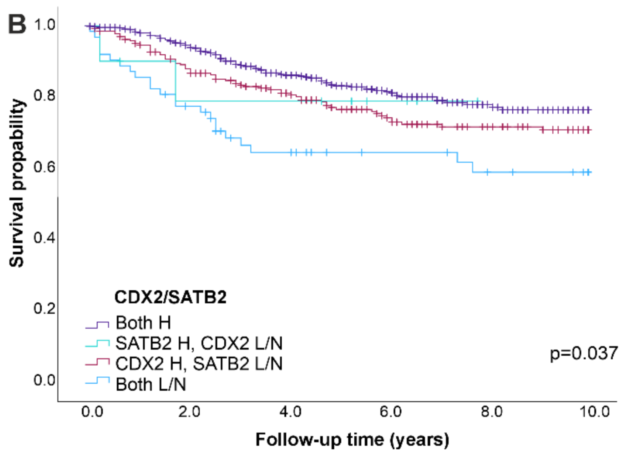

Number at risk

	635	576	383	233	111	65
	10	7	7	3	0	0
	261	223	170	126	94	74
	62	47	32	24	21	16

Figure S4. Kaplan Meier analysis and log rank test of colorectal cancer specific survival according to the combined CDX2/SATB2 expression variable in Cohort 1 (A) and Cohort 2 (B). H High, L/N Low/Negative.

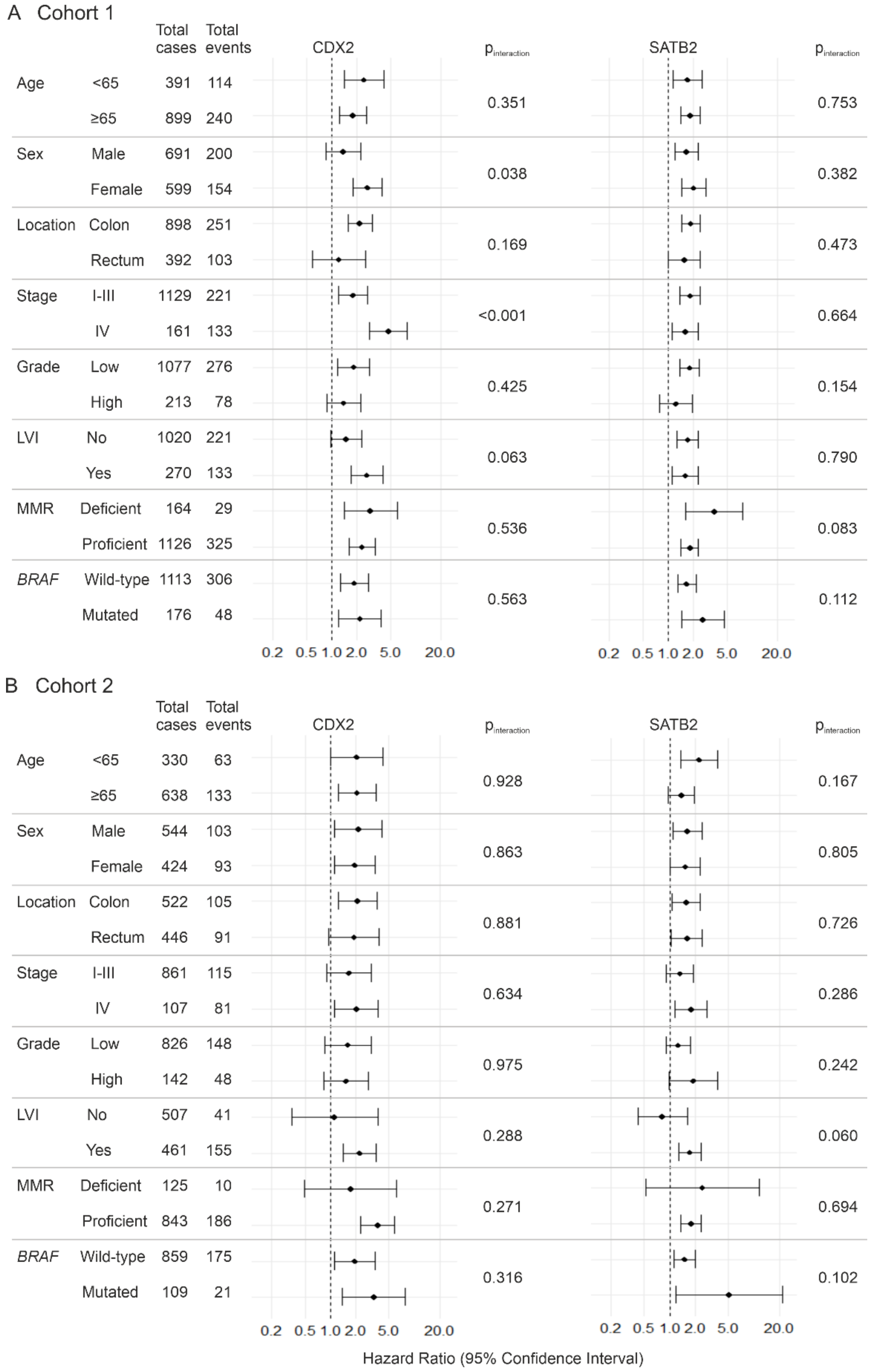

Figure S5. Subgroup analysis of cancer-specific survival by CDX2 and SATB2 expression in Cohort 1 (A) and Cohort 2 (B). The plots present hazard ratios for negative/low (vs. high) CDX2 and SATB2 expression in various patient subgroups. Abbreviations: LVI, lymphovascular invasion; MMR, mismatch repair.
